# Seasonal flow dynamics exacerbate overlap between artisanal fisheries and imperiled Ganges River dolphins

**DOI:** 10.1038/s41598-020-75997-4

**Published:** 2020-11-02

**Authors:** Shambhu Paudel, John L. Koprowski, Michael V. Cove

**Affiliations:** 1 School of Natural Resources and the Environment, University of Arizona, 1064 East Lowell Street, Tucson, AZ 85721 USA; 2 Tribhuvan University, Institute of Forestry, Post Box:43, Pokhara, Nepal; 3 Haub School of Environment and Natural Resources, University of Wyoming, Bim Kendall House, 804 E Fremont St, Laramie, WY 82072 USA; 4 Department of Applied Ecology, North Carolina State University, Raleigh, NC 27695 USA

**Keywords:** Behavioural ecology, Biodiversity, Conservation biology, Freshwater ecology, Socioeconomic scenarios

## Abstract

Here we quantify the effects of artisanal fisheries on the ecology of a small cetacean, the Ganges River dolphin (*Platanista gangetica gangetica*, GRD), in a large river system of Nepal. We examine the size-classes of fisheries’ catches, behavioural changes in GRD in response to fishing activities, and diel overlap between GRD and fishing activity. We observed high human exploitation rates (> 60% of the total catch per effort) of GRD-preferred prey sizes, indicating risks of high resource competition and dietary overlap, especially during the low water season when resource availability is reduced. Competitive interactions in the feeding niches during the low water season, plus temporal overlap between the peak exploitation and critical life-history events (e.g., reproduction), likely have ecological consequences. Furthermore, we detected 48% (95% CI 43–52%) increase in the chance of behavioural changes among dolphins exposed to anthropopressure (fishing activity), risking social behaviour impairment in exposed dolphins. The higher diel overlap and increased diel coefficient as the surveys progressed towards the monsoon season suggest temporal shifts in GRD socio-behavioural states and seasonal effects on resource partitioning, respectively. This work identifies drivers of small cetaceans-fisheries interactions and their consequences, and can be used to help reduce biologically significant fishing impacts on small cetaceans. Mitigation strategies, together with river sanctuary and distanced-based approaches, should be urgently included in a framework of ecosystem-based management.

## Introduction

Freshwater ecosystems provide vital resources for humans and are a habitat for a plethora of endemic and sensitive fauna and flora^1^. However, human pressures on freshwater ecosystems have risen sharply over the past century, leading to substantial and growing threats to global biodiversity^1^. Freshwater fisheries around the world are increasingly overexploited by humans^1,2^. Humans have caused rapid and significant declines in abundance and distribution of freshwater fauna that could reduce future viability of various taxa^2^. Freshwater river systems—exhibiting some of the richest fish biodiversity resources in the world—are no exception to these global trends^3^.

Fisheries in the cold-water systems of the Hindu Kush region of the Himalayas are considered vital sources of nutrition and food security, where large human populations living downstream rely on these natural resources for their daily survival^4^. Large artisanal fishing communities predominantly depend on subsistence and semi-intensive fisheries for their livelihoods. For many Nepalese, fishing is a way of life^5^. Nearly three million fishermen from India and Bangladesh rely on fishing in Himalayan Rivers for income, food security, and nutrition^6^. For example, the Koshi River in Nepal, a major tributary of the Ganges River, harbours 103 fish species and contributes about half of Nepal’s total fish production of 33,000 metric tons per year; with more than 30,000 people depending on fishing in the Koshi and other rivers in Nepal for their livelihoods^6^. Consequently, local dependence on river systems, documenting losses of biodiversity, diagnosing their causes, and finding mitigation have become significant issues.

The first human-caused extinction of a Yangtze River dolphin (*Lipotes vexillifer*) from China has led to growing concern that similar extinctions of other river dolphins are likely unless human needs and river ecology are better understood, and conflicts mitigated^7^. Particularly at risk are other river dolphins with distributions that are mostly restricted to human-dominated river systems under the immense pressure of river-dependent communities. Conflicts between small cetaceans and artisanal fishing have increased globally in recent years^8–12^. Despite available studies on interactions between small cetaceans and fisheries, competition between small cetaceans and subsistence fisheries poses a severe and growing problem, and meaningful management thus requires an effective and appropriate assessment of the factors driving the interaction.

The conflict between small cetaceans and artisanal fisheries is mainly through ecological niche overlap, for example, food and habitats^12–17^. Such interactions have resulted in small GRD population sizes of questionable viability in two major river systems of Nepal, the Karnali and Sapta Koshi^17^. At least 95 Indus River dolphins (*Platanista gangetica minor*) were killed between 1993 and 2012 in fishing gear in the main sections of the rivers of Pakistan^18^. Similarly, the majority of Irrawaddy dolphin (*Orcaella brevirostiris*) deaths were attributed to entanglement in gillnets in the Mekong River of Cambodia and Laos^8^. Furthermore, the survival of South American river dolphins is threatened by fisheries^15^, and the vaquita (*Phocoena sinus*) in Mexico is in severe danger of extinction due to non-target capture in nets^19^. Globally, human activities leading to intense small cetaceans-fisheries interactions must be examined to manage and promote the co-existence between fisheries and small cetaceans.

Previous research has quantified the strength of the interaction between cetaceans and fisheries. However, no studies have examined potential ecological niche overlap, for example, overlap in the dimension of prey size (focusing preferred prey size), diel activity (the distribution of activity throughout the daily cycle), and behavioural distraction (change from one behavioural state to other). Most studies have used fisheries datasets (biological catch) to measure such interactions. For example, by-catch (number of entangled dolphins), harvest details (catch per unit effort), and social dimensions^20–24^ while ignoring the potential relationship between niche overlap and competition^25^.

Globally, a sharp decline in population status (~ 50%) and distribution of GRD is attributed to anthropogenic activities^26^. In Nepal, out of four rivers of distribution, currently only two river systems are occupied by GRD with questionably viable population size (< 50)^17^. Here, we assessed aspects of the interaction between artisanal fisheries and cetaceans in Nepalese waterways not previously studied by examining (1) niche overlap between GRD and fisheries in the dimension of prey size; (2) overlap in diel activity by fisheries and GDR; and (3) effects of fisheries on GDR behaviour. To characterize the interactions, we collected fishery data at wider temporal scale, visually observed GRD response to fishing events, and estimated overlap coefficients to compare activity patterns of fishers and GRD. We analysed the fishery data using generalized linear models within a Bayesian hierarchical framework to obtain exploitation rates of GRD preferred prey sizes and their contributing factors.

## Results

### Preferred prey size exploitation over space and time

The top deviance information criterion (DIC) supported model included gear and season as predictive covariates (Model 1, Table 1). On average, gear and season contribute with β coefficients of 2.438 (95% Credible interval: 2.122–2.743) and 0.193 (95% Credible interval: − 0.121 to 0.502), respectively. The proportion of exploitation (β coefficient) was higher in the Sapta Koshi river [Gill = 0.623 (95% CI 0.45–0.807), Cast = 0.877 (95% CI 0.719–0.955)] than in the Karnali [(Karnali: Gill = 0.521 (95% CI 0.338–0.73), Cast = 0.677 (95% CI 0.518–0.797). Contrary to our a priori predictions, cast nets were associated with higher average exploitation of the proportion of preferred fish size caught during the dry season (October–March), whereas, most of the time (April–November), gill nets were associated with stronger effects.

**Table 1:** Proposed covariates for the model described in Eq. (1), model comparison via Deviance Information Criterion (DIC), effective number of parameters (pD), and Bayesian P-values used to assess model fit for the fishing data.

ID	Model (x_i,t_)	DIC	pD	Bayesian P-value
1	**Gear + season**	**1203.789**	**217.033**	**0.479**
2	Gear + season + gear * season	1203.939	215.142	0.488
3	Gear * season + river	1210.288	215.074	0.515
4	Gear + season + river	1211.893	216.385	0.484
5	Season + time + effort	1214.754	222.301	0.491
6	Season * time	1217.129	222.483	0.5
7	Season + time	1219.104	223.016	0.504
8	Season	1219.676	222.91	0.504
9	Time + effort + distance	1220.401	225.234	0.503
10	Season + time + effort + river	1221.328	221.329	0.51
11	Weight + season	1221.518	222.514	0.5
12	Effort	1222.943	226.32	0.502
13	Null	1223.651	225.923	0.509
14	Weight + season + weight * season	1224.025	222.837	0.516
15	Season * time + river	1225.449	222.444	0.515
16	Weight + season + river	1226.818	222.617	0.508
17	Season + river + time	1227.149	222.9	0.512
18	Time + effort + time * effort	1227.832	226.035	0.516
19	Season + river	1228.121	223.183	0.51
20	Season + river + season * river	1228.513	222.499	0.511
21	Time + effort + river	1228.983	226.851	0.517
22	Season * river	1229.077	223.013	0.513

Models that contained interactions also contained the additive covariate. Among the tested models, model 1 performed best.

Bold values indicate the top deviance information criterion (DIC) supported model.

**Figure 1:**
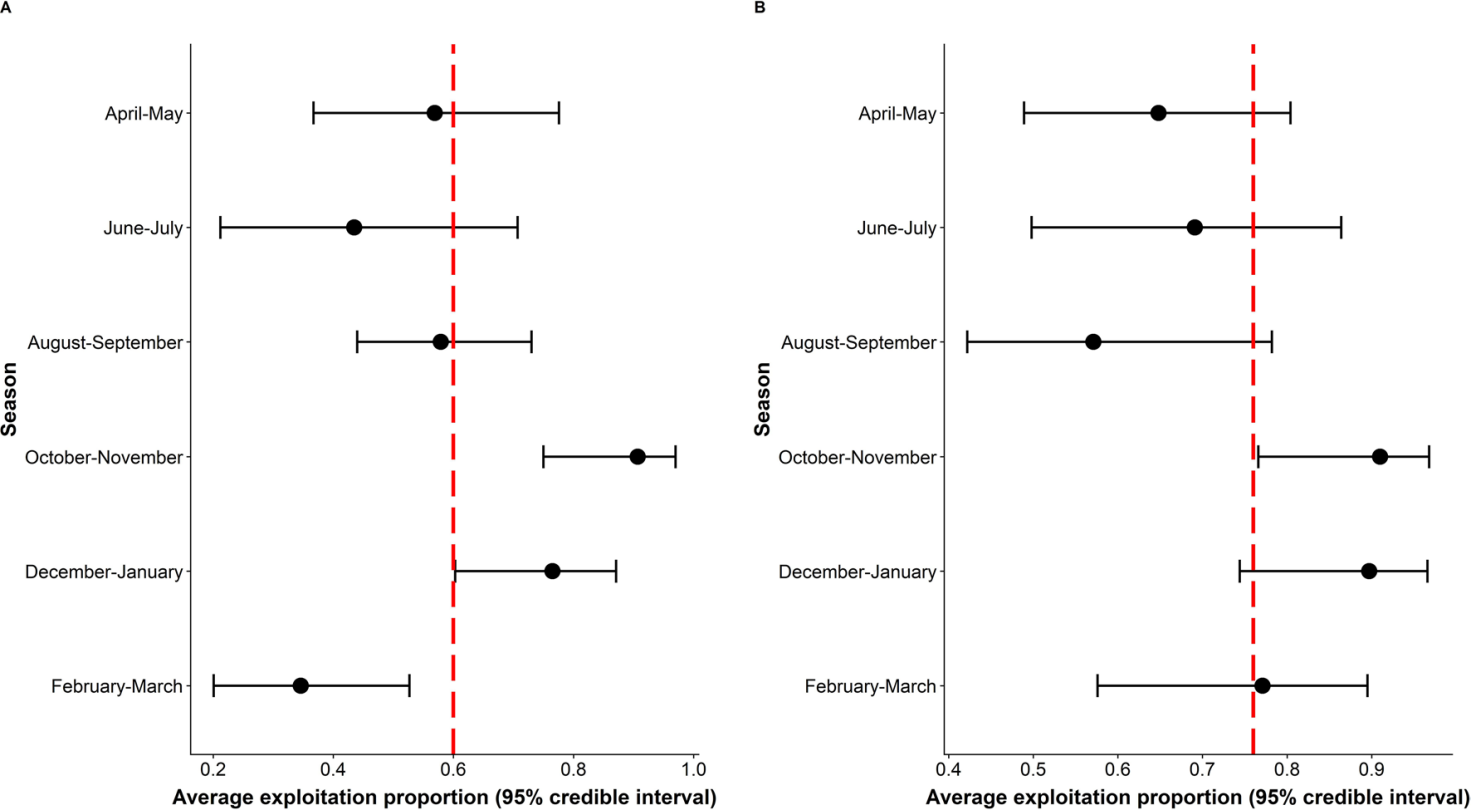
Mean exploited proportion over season, where the red vertical line represents the annual average (**A** Karnali; **B** Sapta Koshi). For both river systems, October–January received a higher amount of exploited proportion, which is greater than the yearly average. The black dot in each error bar represents the mean exploited value for the respective season.

In both river systems, the dry season (October–March) had a higher mean proportion of preferred fish sizes caught compared to other seasons (except February through March in the Karnali River), which were the most common months for the highest percentage of preferred fish size captured (Fig. 1). We detected minimal variation of mean proportional rates during dry seasons, which had the largest mean proportion values compared to other seasons (Fig. 1). The mean annual exploitation proportion rates were 0.6 (95% CI 0.297–0.952) and 0.78 (95% CI 0.732–0.952) in the Karnali and Sapta Koshi rivers (Fig. 1), respectively.

### Fisheries over space and time

**Figure 2:**
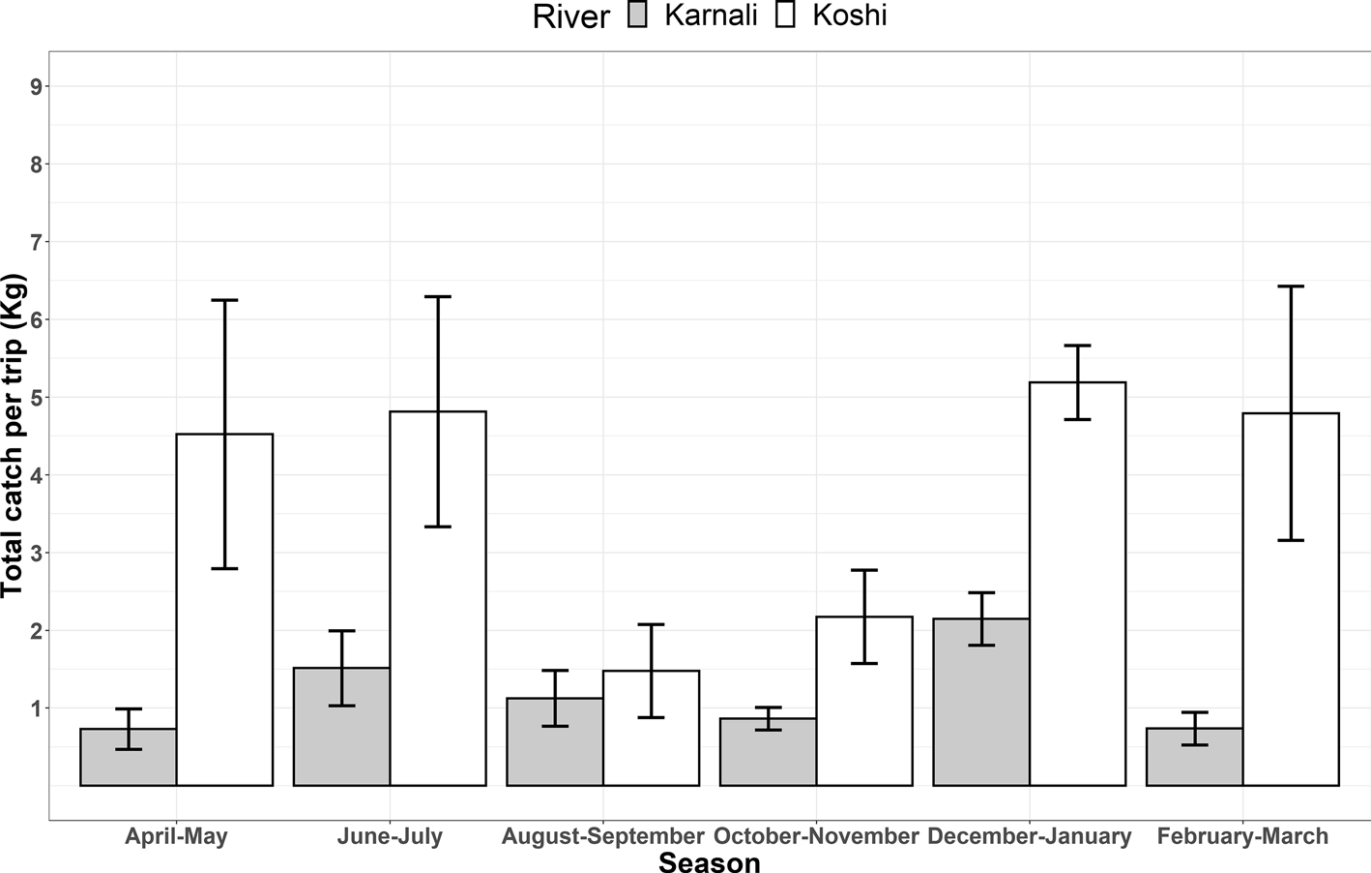
Seasonal average total catches per effort (with 95% CI level) in the Karnali and Sapta Koshi River systems. The Sapta Koshi River shows a twofold higher level of harvest compared to the Karnali River, suggesting a high rate of resource exploitation in the Sapta Koshi river of Nepal. The rate of exploitation is higher from December through July, which is the temporal range with most top fisheries-dolphin conflicts recorded.

**Figure 3:**
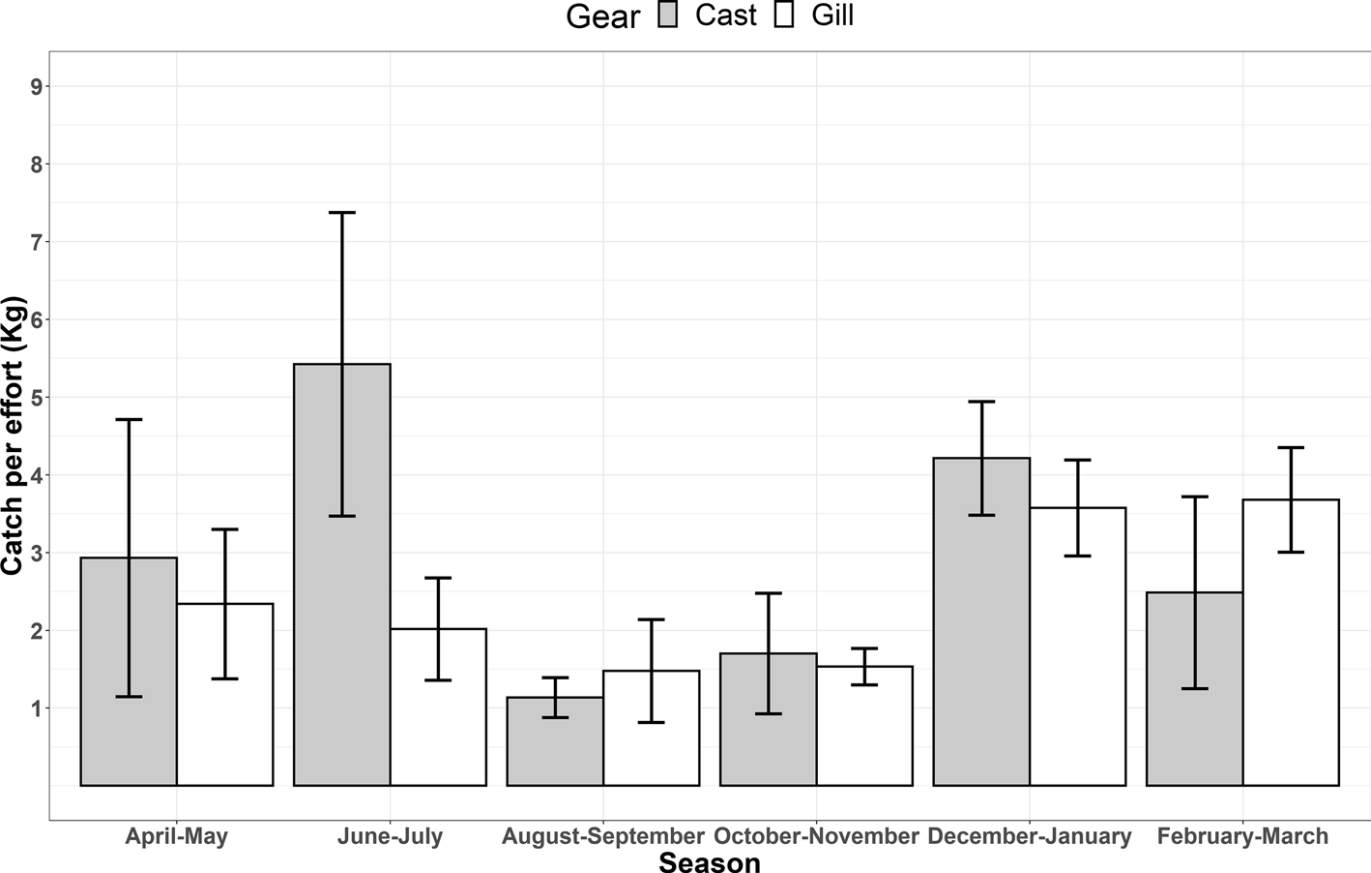
Average catch per effort in terms of gear types (Gill and Cast nets). During the low water season (December–July), the effect of the Cast net is higher compared to the Gillnet, suggesting the significance of the regulation of the cast nets to minimize the resource competition and potential risk of animal depredation.

Average total catch per effort exhibits distinct seasonal patterns in both river systems, lowest in summer (April–November: mean = 1.454 kg, SD = 1.719), and higher during the dry season (October–March: mean = 3.063 kg, SD = 3.236). Across the seasons, June-July (mean = 5.062 kg, SD = 4.620) in the Sapta Koshi and December–January (mean = 2.145 kg, SD = 0.906) in the Karnali attained the highest peaks of fish biomass captured, which then declined to the onset of the summer season. Average total catches varied more widely within a season in the Sapta Koshi River, whereas in the Karnali catches tended to remain constant (Fig. 2). The lowest average catch records were observed through August to November in both river systems, which were less than or equal to 2 kg (Fig. 3). Average catch per effort across gear type [(Cast: Karnali-mean = 0.932 kg, SD = 0.845; Sapta Koshi-mean = 4.470 kg, SD = 4.404), (Gill: Karnali-mean = 1.420 kg, SD = 1.104; Sapta Koshi-mean = 3.141 kg, SD = 2.428)] varied, which considerably contributed to the average seasonal biomass catches observed (Fig. 3).

### Behavioural changes to the artisanal fishing boat

**Figure 4:**
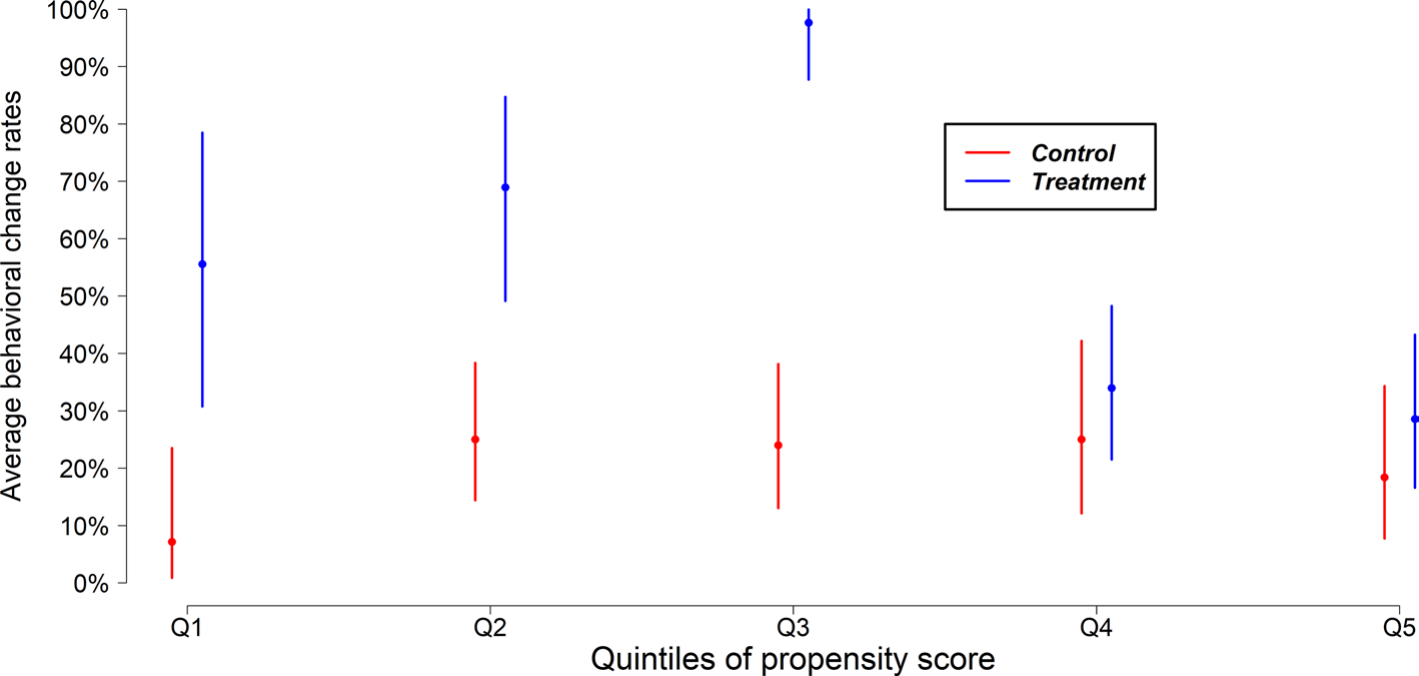
The average behavioural change (overall) percentage by group (treatment and control) within an equal size propensity matching score strata with 95% confidence intervals. Blue and red bars represent treatment (boat presence) and control effects (without fishing boat), respectively. In each pair, the average treatment effect is higher than in the control effect.

Time of the day (GLM, estimate = 0.685, 95% CI 0.526–0.887, Z = − 2.843, P = 0.004) associated with a higher probability of dolphins being subjected to anthropopressure. Compared to the afternoon (estimate = 1.1875, 95% CI 0.7427–1.910), risk of behavioural change in the morning and evening increased by 1.753 (95% CI 1.070–2.879) and 2.470 (95% CI 1.438–4.276) respectively. The propensity scores varied from 0.328 to 0.746 (mean = 0.48), with high overlap of scores between two treatment groups. Behavioural change rates (with 95% confidence intervals) by treatment groups in each propensity quintile group revealed that behavioural change rates were higher in dolphins exposed to anthropopressure than those in the control group (Fig. 4). Overall, we noticed an average 48% (95% CI 43–52%) increase in chance of behavioural change rates (or risks) in dolphins exposed to anthropopressure compared to control group dolphins (Fig. 4). Under a human presence, we noticed substantial change in behaviour state from surface feeding to long (44%, n = 180) and travel dive (33%, n = 135) respectively.

### Temporal activity/diel overlap

**Figure 5:**
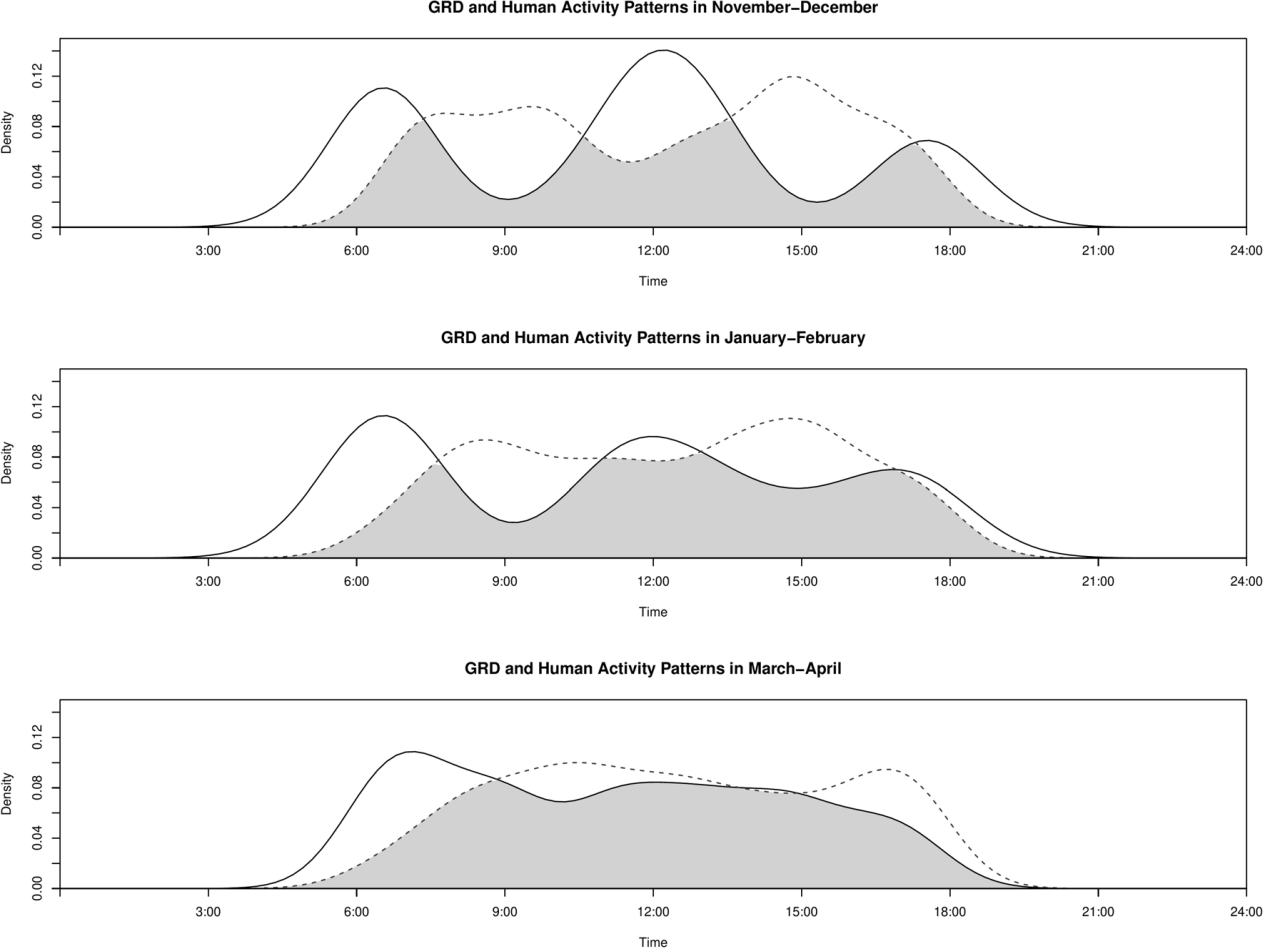
Black lines indicate activities of dolphins, and the humans are blue dotted lines. The overlap increases during each period and from one period to the next (shaded grey), showing a clear trend that there are inverse peaks of activity between dolphins and humans.

We detected GRDs and artisanal fishing boats in all months surveyed, with substantial variation in GRD detections, ranging from 45 and 51 detections in the winter surveys (November–February) to 127 detections during March–April as the monsoon season approached. Artisanal fishing boat detections were fairly constant, ranging from 187 to 195 detections across the same survey periods. Dolphin activity in the dry season (November–February) exhibited trimodal activity curves with a peak in early morning activity around 0700 h, followed by a peak in mid-day (1200 h), and a less pronounced peak towards the end of daylight 1800 h (Fig. 5). Dolphin activity in March–April exhibited similar activity peaks in the early morning (0700 h), but with a distribution of activity that plateaued throughout the rest of the day. Artisanal fishing boat activity across all seasons approached uniformity throughout the day, with minor declines in activity around mid-day. These observations led to moderate diel overlap between the artisanal fishing boats and GRDs in November–December with the coefficient of overlap estimates Δ = 0.604 (95% CI 0.478–0.726, Table 2). Overlap coefficients increased across the next two survey seasons: Δ = 0.725 (95% CI 0.615–0.850) and Δ = 0.832 (95% CI 0.756–0.912) in January–February and March–April, respectively (Table 2).

**Table 2:** Coefficient of overlap (∆) estimates and 95% confidence intervals of the diel activity patterns of Ganges River dolphins (GRD) and humans during three sampling periods: November–December, January–February, and March–April in the Sapta Koshi River, Nepal.

Period	Number of detections		Coefficient of overlap (∆)	Lower CI	Upper CI
	GRD	Humans			
November–December	51	189	0.604	0.478	0.726
January–February	45	187	0.725	0.615	0.850
March–April	127	195	0.832	0.756	0.912

## Discussion

Globally, interactions between artisanal fisheries and small cetaceans are one of the most significant conservation issues leading to endangerment and extinction^27^. The recent extinction of the Yangtze River dolphin and the critically small size of the vaquita population showcase how such interactions can contribute to dramatic declines of small cetaceans^18^. Our study reveals that interactive and cumulative effects of artisanal fishing with seasonal resource variations—the high dietary overlap of preferred prey exploitation, substantial risk of impairment of ecological and social behaviour, and significant diel activity overlaps—is putting the small cetaceans under acute pressure that could affect dolphin persistence. These factors might have contributed to the decline in dolphin populations in Nepal over the past two decades^12^ and will continue to affect river dolphin population growth in the future.

We observed high human exploitation rates of GRD-preferred prey sizes in the major river systems of Nepal, where an already small number of the dolphins (28–52 individuals) persist^12^. Greater than 60% of the total catch per effort in each river was within the preferred prey size range. Fishing gear and seasons were identified as significantly contributing to the higher proportion of GRD-preferred fish size-classes captured in fisheries. Among these contributing factors, the contribution of gear was twofold stronger than that of season. Specifically, we saw a higher proportion of GRD-preferred fish caught by cast nets relative to gill nets, particularly during the dry season. Because of non-selective behaviours of the cast net, impacts of cast nets could be significant relative to gill net during dry seasons when resource availability is reduced. Medirose et al.^28^ reported similar effects of cast nets in aquatic systems of Brazil and urged fishing gear specific fisheries management schema. As the variation on fish stock and size distribution is likely regulated by fishing and temperature (regulated by season), seasonal variation in resource overlap highlights seasonal gear-specific regulations as the appropriate and effective mitigation effort to sustain resources and minimize potential conflict between small cetaceans and artisanal fisheries in highly exploited river system.

Also, these high exploitation rates peaked during the dry season when habitat was reduced, which suggests that there is typically more significant overlap in the feeding “niches” of river dolphins and fishermen during this time of year. Narrow habitat breadth coupled with limited availability of habitat and overlap in prey resources likely increases competition between GRD and fisheries in that both GRD and artisanal fisheries overlap in high productivity foraging sites, such as deep pools and confluence habitats^29^. The direct effects of these interactions have likely increased by-catch of small cetaceans. For example, we recorded by-catch leading to the death of three young calves in the Sapta Koshi River and one adult in the Karnali River during 2015–2018, especially between December–February. Such mortality could be attributed to either small cetaceans increasing their foraging time in the area with high overlapped, which forces small cetaceans to forage further in areas with higher depredation risks^30^. Although fish availability or stock data is not available for the study site, the high exploitation of small cetacean’s preferred prey by fisheries during the resource-limitation period suggests there is high overlap between the diets of small cetaceans and the catch of artisanal fisheries, which might play an indirect role in compromising the health of these cetaceans^31^. We did not find any species that are devalued by the fisheries, but we noticed fisheries opting for a few species in terms of taste. Fishers directly sell these captured fishes in a local market, where fishes are consumed in multiple ways.

Furthermore, we noticed GRD reproduction predominantly occurs during the low water season when fishery exploitation is highest. Limited habitat availability, along with the risk of phenological mismatch between predator and prey in the changing Anthropocene, further exacerbate the risk of population decline or extirpation in small cetacean (such as GRD) by adversely affecting reproduction^32^. For instance, the loss of four small cetaceans in the Mediterranean and Black Seas has been attributed to overfishing or prey depletion^33^.

It would be difficult to establish a clear link between fisheries and the decline of dolphins. Nevertheless, under the lack of the most preferred food or prey size could play a significant role to reduce potential reproduction, and is considered as the most important population size regulator^33^. Thus, small cetaceans are highly vulnerable to such competition effects, which likely affects their survival, fecundity, and overall fitness^34^.

We observed a difference in the behavioural change rate of GRDs between anthropopressure and control zones. A chance of higher dolphin behavioural change rates (40–60%) were observed in artisanal fishing boat areas compared to control areas. Although there is no direct evidence of the adverse impact of such behavioural changes on small cetaceans’ ecology, we suspect that artisanal fishing activities might threaten individual social or behavioural roles that are essential to maintain a cohesive functioning society^18^. Chilvers et al.^35^ also noticed the change in the behaviours of the bottlenose dolphin communities (*Tursiaps truncaría*) as a function of fishing activity. Disruption of the behaviours and social life of Indo-Pacific humpback dolphins (*Sousa chinensis*) by fishers is argued to be a form of short-term stress that leads to a permanent impairment of behavioural functioning and social life^36^. Thus, among small cetaceans, changes in behaviours, direction and speed of travel, and diving styles (short, long) were all common consequences of such interactions^37^, possibly reducing survival and reproductive success of individuals and declining populations size over time^38^.

Furthermore, distraction in surfacing ecology might reduce energy reserve, which also accelerates physiological stress^39^. While the influences of human-made noise and other human actions on cetacean behaviour have been widely highlighted, cases of increased sensitivity following harassment are emerging in the field of small cetacean conservation^40^. Additional work is needed to determine if the observed behavioural changes we noted in GDR due to fishing activities result in reduced survival. Increasing human-related activities like fishing pressure and changing environment could potentially reduce the space by eliminating access to suitable foraging sites and further fuel physiological harassment. Therefore, possible regulations, including buffer zones or a river sanctuary covering small cetacean hot spots, could reduce both chronic and acute interactions between artisanal fisheries and small cetaceans.

Globally, a lack of information exists on the “area of influence” within which human activities might displace small cetaceans. In line with the findings of Richman et al.^41^, we suggest that 400–500 m can be considered as the maximum GRD observable distance. Furthermore, our maximum response distance (200 m) could be a guide to determine whether small cetaceans experience stress from human activities, and inform co-existence management plans and buffer zones for critical hotspots or observed individual dolphins. The National Oceanic and Atmospheric Administrations of the United States (NOAA-Fisheries) prohibit approaching or remaining 460 m of an endangered Atlantic right whale (*Eubalaena glacialis*), and the distance is considered as a safe distance to enhance their self-sustaining population. Thus, our proposed distances support a strategy to identify specific areas that are important to the survival of small cetacean populations and restricting human access^42^. A recent study^24^ suggests a spatial approach as a cost-effective tool for reducing all fishery-cetacean interactions. In light of this evidence, our response distance might be cost-effective and allow managers to prioritize locations and apply different management strategies, offering potential beneficial outcomes for small cetaceans and fisheries.

Given that many biological activities are under photoperiodic control, allowing very narrow periods for critical life-history events within the annual cycle^43^, shifts in the timing of the essential ecological and social states caused by the diel overlap or human disturbances might have significant fitness consequences in small cetaceans^44^. We noticed a high diel overlap between fisheries and GRD behavioural events, and as a consequence, GRD are displaced from their active surfacing time window. As a result, the optimal timing of critical life history activities that are based on environmental cues, for example, the timing of reproduction, the timing of hibernation or resting, and accumulation of body reserve could be severely affected and thus, reduce the fitness by failing to respond optimally to time-sensitive behaviours^45^. Specific biological effects of timing shifts in small cetaceans have not yet been explored, but broadly deleterious effects have been noted that might influence reproduction success, health, ranging patterns, and availability of preferred habitats, and potentially trigger a decline in abundance^46^. Given the recent human-caused extinction of Yangtze River dolphin^7^ and Mediterranean striped dolphins in 1990–1992^31^, it is likely that small and isolated sub-populations of cetaceans will be severely affected by temporal activity displacement, leading to adverse impacts on the processes that retain demographic dynamics. These might include river dolphins in South and East Asia (e.g., Indus River dolphin, GRD, Irrawaddy dolphin) and some species [e.g., Bolivian River dolphin (*Inia geoffrensis boliviensis*), Chilean dolphin (*Cephalorhynchus eutropia*)] in South America.

Knowledge of temporal activity patterns should improve understanding of surfacing or foraging strategies of small cetaceans and further help to minimize potential conflicts between cetaceans and fisheries^47^. Our temporal activity overlap analysis between GRD and artisanal fisheries revealed relatively high diurnal overlap during the dry seasons (particularly between November to April). The overlap coefficients show an increasing rate as the season gets closer to the onset of the monsoon, suggesting more competition (including spatial overlap) during the post dry season (or pre-monsoon period: February–May). There appear to be inverse peaks in activity between GRDs and artisanal fishing, suggesting that small cetaceans might be temporally avoiding peaks in artisanal fishing activity. We visually observed three distinct temporal fishing patterns adopted by the fishermen: early morning (0530 h), early afternoon (1300 h), and late evening (1600 h). Thus, dolphins exhibit clear peaks in the early morning (0600–0900 h), around noon (1100–1300 h), and late evening (1700–1800 h), suggesting that management plans implementing active time windows might help to minimize long-term effects on small cetaceans. In the past, most small cetacean management efforts have focused on the number of individuals (or by-catch) and have not considered behavioural ecology in management schemes. Our study shows that regulating fishing activity using small cetaceans surfacing ecology as a basis further helps to reduce the adverse effects of human activities. For example, in our case, restricting fishing activity in the early morning (0300–0900 h) and later afternoon (1500–1900 h) could reduce dolphin and artisanal fisheries temporal conflict by ~ 40% and promote co-existence of GRD and fisheries in highly fragmented waterways. However, further understanding of underwater behaviours or feeding strategies of small cetaceans in relation to fishing^48^, body nutritional condition^49^, and examining temporal dynamics of catches^50^ will help to more precisely understand the extent and level of interaction.

Globally, a major threat to small cetaceans (and their subspecies) is their interactions with fisheries, either directly or indirectly, which put them in danger of extinction^51^. Careful monitoring and regulation of the artisanal fisheries, including the development of river sanctuaries, are generally essential for providing sustainable benefits to both small population cetaceans and communities^52^. Given the global demise of small cetaceans, existing conservation policies and structure of protected areas still do not afford sufficient protection for small cetaceans from disturbances. For example, in Nepal, conservation policies and establishment of protected areas are entirely based on terrestrial species, and there have been no river sanctuaries established to date to address the issue of aquatic species and safeguard their migratory pattern. The Vikramshila Gangetic dolphin sanctuary in India is one promising conservation effort to recover the GRD population along the continuum of the Ganges River. If the establishment of protected areas is not feasible, using the GRD’s maximum observable or response distance supports to establish the distance-based regulations between the boats and the dolphins for no interactions. Thus, recovering viable populations of small cetaceans into their natural habitat is potentially costly due to overlap with human economies in the changing environments. Improving and managing fisheries activities is a feasible and cost-effective approach to minimize conflict; however, incorporating river ecology and cetacean behaviour when formulating a management plan is critical. Thus, further understanding of GDR and other small cetacean diel activity patterns (e.g., via noninvasive surveys throughout the 24-h clock) is required to understand life-history strategies and their requirements, thus supporting efforts to maintain co-existence of fishers and small cetaceans. Managing fisheries should not be limited to satisfying consumers, and should incorporate a wide array of ecological and social benefits^53^. Unregulated fisheries practices threaten not only small cetaceans but also the ecology of the rivers and the biotic communities that rely on them. Thus, setting up the appropriate institutional structure and practical legal framework that allows stakeholders to participate in resource management activities is essential for the successful implementation of artisanal fisheries management. Such approaches have demonstrated some success in fisheries management in Brazil^54^ and China^55^. Similarly, Dewhurst-Richman et al.^56^ highlighted the importance of institutional regulations to minimize by-catch of GRD in Bangladesh. Given the burden that freshwater systems are experiencing, peer-based off-farm group activities (e.g., aquaculture in ponds), combined with economic incentives using locally available resources and creating cetacean-based market (eco-tourism), should be included within a framework of integrated river basin management.

## Methods

### Study sites

We conducted this study in two river systems (Sapta Koshi and Karnali) of Nepal (see^5,12^ for geographic distribution, and socio-economic and ecological description of study sites), where the last GRD populations remain^12^. Both river systems represent the upstream range for the GRD distribution in the Ganges River basin^26^. The high dependency of local fishers (78.5%)^5^ on these river systems corresponds with heightened fishing intensity and as a result, deep pools which are most preferred by dolphins occupied by fisheries that reduce habitat availability to dolphins, particularly during the dry seasons (October–March) when flow reduced. Though systematic data on the total number of local fishers depending on river systems is not available, a high proportion of the people living close to river systems derive income primarily from the fishing. Greater than 70% of fishers fish more than 4 days per week in these two river systems using a wooden boat comprised of two boat passengers^5^. The Karnali and Sapta Koshi Rivers provide 55 km and 35 km of potential dolphin habitat, respectively. However, this habitat shrinks considerably during the dry season when available space is reduced, and pressure from fishers escalates^5^.

### Data collection

#### Dolphin preferred prey size exploitation

Since niche overlap (either diet or space) is considered as a prerequisite of competition, interactions should be understood in connection with the niche concept^25^. Diet overlap (in terms of fish size class) could serve as an effective indicator of current interaction levels^57^. We examined the abundance of dolphins’ preferred prey size in the total catch per effort to predict the current direct competitive interactions between GDR and fisheries and the pressure of fisheries on the feeding habits of river dolphins. A catching effort to each net type was defined as an average duration of effective fishing activity of a single trip, excluding travel time (Cast: n = 203, average fishing duration = 3.95 h, SD = 2.04; Gill: n = 198, average fishing duration = 6.68 h, SD = 3.90). Mean catch per effort for each gear type, and average total catch per trip (combined data) by season and river were estimated.

Specific fish sizes have been previously reported as GRD-preferred prey regardless of species^10,52,58^. We considered fish in the range of 3–15 cm total length as preferred fish size regardless of species. We sampled across six 2-month temporal sampling periods between April and March in 2017–2018. We collected 30 landing observations (equal sample size across seasons and between rivers) representing catches per trip for each sampling period. These temporal periods were differentiated to account for potential seasonal variations in fishing stocks. We approached fishers randomly at landing sites immediately before they sold their daily catch of fish. Fish were caught using cast and gill nets during the morning and late afternoon, which are the dominant fishing strategies in both the Karnali and Sapta Koshi rivers. To improve the precision of the estimate, we further stratified landings based on gear types (cast or gill net) and time of the day [morning (0500–1100) or evening (1500–1900) shift] they fished. From the total fish caught, we placed fish greater than 2 cm in total length in a temporary holding tank and then randomly selected ~ 20–100% of the fish to be measured. We recorded fisheries biological information with fishers at landing sites per event [fish total length or size (cm), total fish caught (kg), distance travelled while fishing (km), gear type, and time of the day they fished their gear]. All fishers survey procedures were carried out in accordance with the ethical standards of the Human Subjects Protection Program at the University of Arizona. Informed consent was obtained from all fishers for being included in the study. All the experiment protocols involving humans were approved by the University of Arizona, Institutional Review Board committee charged with the protection of human research subjects. All methods involving fish and dolphin observations were carried out in accordance with the Department of National Parks and Wildlife Conservation, Government of Nepal, guidelines and regulations. Fish measurement protocols, including dolphin observation methods, were approved by the Department of National Parks and Wildlife Conservation, Government of Nepal (No 1129; 12 December 2016).

#### Behavioural responses to fishing events

We examined behavioural responses of river dolphins to artisanal fishing boats as an index of disturbance. Here we defined “behavioural response” as any alteration of behavioural state (from one to another state) as a putative consequence of interacting with a fishing boat. Because of immense threats from fisheries to GRD survival (i.e. entanglement) in the Sapta Koshi^12^, we observed behavioural states from November through May during 2017–2018 only in the Sapta Koshi River of Nepal. This temporal window represents the dry season, in which conflicts between dolphins and fisheries is heightened as a function of reduced deep pool habitats^17^. As artisanal fisheries in Nepal primarily use only unpowered wooden boats (absence of engine boat or any other heavy commercial traffic vessels) and adopt spatial partitioning of fishing activities (isolated from other fishers) among fishers to avoid potential fishing competition, this assisted us to record behavioural response to each fishing activity. If multiple fishing boats were present or travelling through the area, we excluded such observations from the study. Assuming < 500 m as the maximum observation distance of GRD^41^, we divided the river into shore-based transects of 400 m, with one elevated fixed observation station (3 m from the river bank to avoid possible disturbances) in the centre of each transect (at 200 m). This allowed us to classify the dolphin-fisheries interaction points into pressure (treatment) or control zones. We classified the dolphin presence area into control (without boats, control group) or pressure (presence of fishing boat, treatment group) zones using the maximum dolphin response distance to a fishing boat. To classify the zones, we defined maximum dolphin response distance as 200 m (SD 25 m, n = 156); this decision was based on the findings of a pilot study. We conducted a shore-based pilot survey to estimate the approximate response distance, with a distance < 200 m from the fishing boat considered as anthropopressure treatment, and > 200 m classified as the control zone. We classified dolphin behaviour into five states that we could distinguish at a distance from these observation stations: [Dive (D), steep dives with long dive interval showing tails out at the surface before the dive; Travel (T), persistent and directional movement with constant speed and relatively short dive intervals (< 60 s); Surface-Feeding (SF), chase fish with rapid circular dives, rapid directional changes, and circle swimming; Socializing (S), engage in diverse interactions with some physical contact with other dolphins; and Resting (R), low swimming speed with short dive intervals and no group activity].

We applied focal animal scan-sampling surveys as the strategy to record sequential behaviours, but if dolphins were observed together, we considered these groups as a single analysis unit. When a dolphin was first sighted, we classified the initial behavioural state and the behaviour exhibited at the end of a 3-min time interval following the initial sighting. Any individual that disappeared before the 3 min period expired was excluded from analyses. When dolphin was sighted in a group, we recorded the predominant behaviours observed in the group (usually each member engaged in similar behaviour patterns). We recorded a total 406 behavioural observations (n) throughout the season, representing evenly distributed (n = 67–68) observations across the months. Out of the total observations, 208 observations were in the control zone, and 198 were in the pressure zone.

### Data analysis

#### Preferred prey size exploitation by fisheries

We used generalized linear mixed models within a Bayesian hierarchical framework to quantify the proportion of catch by fishers that fell within the preferred prey size of dolphins (i.e., the proportion of all fish caught that were within 3–15 cm). The model structure followed:

1 $${Y}_{i,t,j }\sim \mathrm{Binomial} \left({n}_{i,t,j} , {p}_{i,t}\right)$$

$$logit\left({p}_{i,t}\right),\sim \mathrm{Normal} \left({{X}^{^{\prime}}}_{i,t}\beta ,{\sigma }^{2}\right)$$

$${\beta }_{,} \sim \mathrm{Normal} \left(0,{\sigma }_{\beta }^{2}\right)$$

$${\sigma }^{2} \sim \mathrm{IG }\left(a,b\right),$$

$$\sigma { }_{\beta }^{2}\sim \mathrm{IG }\left(c,d\right),$$

where y_i,t,j_ is the number of fish caught that were between 3 and 15 cm at site i = 1,…n, during time period t = 1,…T, for boat j = 1,…J. We represent the total number of fish caught at site i by boat j during time t with n_i,t,j_. We were interested in estimating the expected proportion of total fish caught that were between 3 and 15 cm, p_i,t_, given the characteristics of the site described by covariates in the vector x_i,t_. The β coefficients characterize the direction and strength of the relationship between the covariates x_i,t,_ and the logit of the proportion of fish between 3 and 15 cm. We developed a suite of a priori predictive models to describe differences in proportions p_i,t_ among sites and times (e.g., different combinations of covariates in the vector x_i,t_, Table 1). We assessed the relative strength of these hypotheses by fitting Eq. (1) to our data for each x_i,t_ in Table 1, and calculating the Deviance Information Criterion (DIC) for each model^59^. We used vague priors for all parameter distributions. Specifically, we used inverse gamma (IG) priors for σ^2^ and σ^2^_β_ and set a = b = c = d = 0.0001. We fit each model to our data using a custom Markov chain Monte Carlo algorithm written in R statistical software^60^. For each model fit, we obtained two chains of 50,000 iterations after a suitable burn-in period. We assessed chains visually and also used the Gelman-Rubin convergence diagnostic. All chains appeared to converge and had Gelman-Rubin diagnostic values < 1.01. We further assessed model fit using Bayesian P-values^61,62^. We used a random effect for each location and time (i.e., line 2 in Eq. 1) because preliminary analyses suggested that a generalized linear model without a random effect had extreme lack of model fit (i.e., Bayesian P-value = 1). After incorporating the random effect, we found no evidence for lack of model fit for any model we considered (Table 1). Furthermore, we estimated the posterior distributions for each of these covariates using a Beta posterior distribution function, with the binomial distribution as a sampling distribution type. We simulated 10,000 random samples from this posterior distribution taking a uniform Beta before summarizing results for each gear type and season.

#### Behavioural changes to artisanal fishing boats

We applied propensity matching score analysis (PMS, conditional probability) to estimate the causal effects of treatment (presence of fishing boat) on GRD behaviour change^63^. As our observations were non-random, we used PMS to mimic the conditions of a randomized controlled trial using all the information (covariates) sufficient to predict the probability of receiving the treatment effect. Thus, in PMS, the likelihood that each observation gets a particular treatment effect is the same for all observations (either control or treated), such that they depend only on the known explanatory variables of an observation. Targeting fishing activity as the primary factor, other than natural processes, for behavioural distraction, we included seasons (month) and time of the day [morning (400-11:59), afternoon (1200-14:59), evening (1500-1900)] as additional explanatory variables. As we are concerned only with the fishing activity, we assumed that these covariates are sufficient to predict the effect of the treatment. To estimate the propensity score, we used a Generalized Linear Model (GLM) using logit link function in which treatment status (treatment vs. control environment) regressed on the baseline characteristics of seasons (month) and time of the day. We compared four a priori models with the explanatory variables [model1: time of the day, model2: seasons, model3: time of day + seasons, model4: time of day * seasons] and used the model with the lowest AIC value (Akaike information criterion) to predict the effect of the treatment. The model with the covariate time of the day (model1-AIC 549.640; model2- AIC 555.585; model3 AIC-550.024, model4-AIC 549.996) was best fitted to estimate the effects of explanatory variable on the probability of receiving treatment (e.g., anthropopressure-fishing boat). We forwarded this model to estimate the propensity scores. To ensure that our observations were randomly assigned, we visually checked propensity scores overlap between two treatments and found a good degree of overlap (between 0.32 and 0.78). Furthermore, to ensure that our covariate (time of day) provides sufficient information to tell the dolphin’s probability of receiving the treatment, we visually checked the balance of covariate across treatment groups by stratifying propensity matching scores into equal-sized quintile strata (Q1, Q2, Q3, Q4, and Q5)^64^. We examined the distribution of covariate within the quintiles of the propensity scores and observed that the distribution of propensity scores closely aligned (no major differences) between treatment groups for explanatory variable. The balanced covariate within quintiles of the propensity score gives an opportunity to estimate unbiased treatment effect (fishing boat presence) within each propensity score stratum^65^. Thus, we estimated the average treatment and control effects in each propensity score quintile and also derive the overall probability of receiving the treatment. All statistical analysis was done in R and PMS estimated using package ‘matching*’.*

#### Activity overlap analyses

We used the behavioural response (time of dolphin first detection) and fishing (time of fishing activity lasting ≥ 10 min) event observational datasets to analyze the diel activity overlap between fisheries and river dolphins. We treated the times of dolphin behavioural observations and the times of actively engaged fishing with cast or gillnet observations as random samples from the continuous distribution of the 24-h clock. However, our inferences were limited to the diurnal portion of the day (0600–1900 h), because we did not make observations at night and fishing activity was mostly absent during night hours. We used the kernel density overlap approach to fit a von Mises kernel density to each activity distribution for dolphins and humans^66^. We calculated the coefficient of overlap (Δ) as the proportion of diel overlap between activity patterns of dolphins and humans, which bounded between 0 (no overlap) and 1 (complete overlap)^66^. We estimated the overlap coefficients for bimonthly periods to account for seasonal variation in these activity patterns and to ensure robust sample sizes for inferences^67^. We performed 10,000 iterative bootstraps to determine the 95% confidence intervals within the ‘overlap’ package in R to account for uncertainty in our estimates^68,69^.

## Data Availability

All data supporting the conclusions of this article are within the paper.
